# Rapid Pathogen-Induced Apoptosis: A Mechanism Used by Dendritic Cells to Limit Intracellular Replication of *Legionella pneumophila*

**DOI:** 10.1371/journal.ppat.1000478

**Published:** 2009-06-12

**Authors:** Catarina V. Nogueira, Tullia Lindsten, Amanda M. Jamieson, Christopher L. Case, Sunny Shin, Craig B. Thompson, Craig R. Roy

**Affiliations:** 1 Section of Microbial Pathogenesis, Yale University School of Medicine, New Haven, Connecticut, United States of America; 2 Graduate Program in Areas of Basic and Applied Biology, Instituto de Ciências Biomedicas Dr. Abel Salazar, Universidade do Porto, Porto, Portugal; 3 Department of Cancer Biology, Abramson Family Cancer Research Institute, University of Pennsylvania, Philadelphia, Pennsylvania, United States of America; 4 Department of Immunobiology, Howard Hughes Medical Institute, Yale University School of Medicine, New Haven, Connecticut, United States of America; University of Washington, United States of America

## Abstract

Dendritic cells (DCs) are specialized phagocytes that internalize exogenous antigens and microbes at peripheral sites, and then migrate to lymphatic organs to display foreign peptides to naïve T cells. There are several examples where DCs have been shown to be more efficient at restricting the intracellular replication of pathogens compared to macrophages, a property that could prevent DCs from enhancing pathogen dissemination. To understand DC responses to pathogens, we investigated the mechanisms by which mouse DCs are able to restrict replication of the intracellular pathogen *Legionella pneumophila*. We show that both DCs and macrophages have the ability to interfere with *L. pneumophila* replication through a cell death pathway mediated by caspase-1 and Naip5. *L. pneumophila* that avoided Naip5-dependent responses, however, showed robust replication in macrophages but remained unable to replicate in DCs. Apoptotic cell death mediated by caspase-3 was found to occur much earlier in DCs following infection by *L. pneumophila* compared to macrophages infected similarly. Eliminating the pro-apoptotic proteins Bax and Bak or overproducing the anti-apoptotic protein Bcl-2 were both found to restore *L. pneumophila* replication in DCs. Thus, DCs have a microbial response pathway that rapidly activates apoptosis to limit pathogen replication.

## Author Summary

The immune system is designed to identify microbes that enter the body and elicit responses that prevent the replication and dissemination of these organisms. Dendritic cells play an important role in regulating host immunity to pathogens. Their phagocytic capacity enables DCs to internalize and destroy most microbes, and the ability of DCs to migrate to specialized lymphoid organs is important for inducing antigen-specific immunity. Here, we analyzed interactions between DCs and *Legionella pneumophila*, a bacterial pathogen that can subvert phagocytic host cell functions to create a vacuole that permits intracellular replication. We found that *L. pneumophila* infection rapidly induced DCs to commit cell death through apoptosis. Rapid apoptosis was not observed after infection of macrophages, which are the phagocytic cells that support *L. pneumophila* replication in the lungs of infected animals. Using cells derived from knockout mice, we found that DCs deficient in the proteins Bax and Bak, which are essential for induction of the apoptosis pathway, were unable to restrict the intracellular replication of *L. pneumophila*. Likewise, overproduction of Bcl-2, which is a negative regulator of apoptosis, resulted in DCs that were permissive for *L. pneumophila* replication. These data indicate DCs have the ability to rapidly undergo apoptosis when infected with a microbe capable of replicating intracellularly, and this response effectively prevents pathogen replication. We hypothesize that this response may be designed to interfere with the migration of infected DCs through the lymphatic system, which would prevent DCs from serving as a “Trojan Horse” that transports pathogenic microbes from peripheral sites to central organs.

## Introduction

Macrophages and dendritic cells (DCs) are the sentinels of the innate immune system. They are key in sensing infection and activating downstream antimicrobial responses [1],[2]. These professional phagocytes are activated following stimulation of pattern-recognition receptors, such as transmembrane Toll-like receptors (TLRs) and cytoplasmic nucleotide-binding domain and leucine-rich repeat containing receptors (NLRs) by pathogen-associated molecular patterns (PAMPs). Signaling through these receptors induces the expression and secretion of proinflammatory cytokines, chemokines and other antimicrobial defense molecules [3]–[7].

Bacterial pathogens that are able to infect and establish residence within macrophages and DCs provide a unique challenge to the innate immune system, as many pathogens have evolved virulence factors that subvert the cellular processes of these cells. One such pathogen is *Legionella pneumophila*, the etiological agent of the severe pneumonia known as Legionnaires' disease [8],[9]. *L. pneumophila* is able to infect alveolar macrophages and modulate transport of the phagosome in which it resides to avoid fusion with endosomes and lysosomes [10]. *L. pneumophila* has the ability to recruit vesicles in transit between the endoplasmic reticulum (ER) and Golgi apparatus and use these vesicles to remodel the *L. pneumophila*-containing vacuole (LCV) to create a unique ER-derived vacuole that supports intracellular replication [10]–[17]. Modulation of intracellular transport of the LCV requires a functional type IV secretion system (TFSS) encoded by the *dot* and *icm* genes, which translocates bacterial effectors directly into the host cytosol [18]–[21]. Many of the translocated effector proteins engage host factors involved in vesicular transport and assist in LCV transport [22]–[29]. *L. pneumophila* mutants defective in the Dot/Icm system do not replicate intracellularly, as they are unable to modulate intracellular transport and occupy a more conventional phagosome that undergoes rapid endocytic maturation [19],[30].

Although *L. pneumophila* has evolved sophisticated strategies to overtake phagocytic host cells, the mammalian innate immune system is able to efficiently control bacterial infection and replication. Responses controlled by the TLR adaptor protein MyD88 effectively clear *L. pneumophila* from the lungs of infected mice [31]–[33]. NLRs also contribute to the detection and control of infection. Naip5 (Birc1e), an NLR encoded within the *Lgn*1 locus, limits replication of *L. pneumophila* in mouse macrophages [34]–[37]. Naip5 is activated by a Dot/Icm-dependent signaling event that presumably involves the delivery of the bacterial protein flagellin into the host cell cytosol [38]–[40]. Naip5 in conjunction with the NLR protein Ipaf activates caspase-1, which limits *L. pneumophila* replication in macrophages by inducing a pro-inflammatory cell death pathway known as pyroptosis [37]–[40].

Naip5 control of caspase-1 activation does not seem to be the only cellular mechanism used by innate immune cells to control *L. pneumophila* replication. In DCs infected with *L. pneumophila*, although phagosomes containing bacteria are able to mature into ER-derived organelles, bacterial replication is limited [41]. DCs are still able to process and present *L. pneumophila* antigens on MHC class II molecules, and *de novo* synthesis of *L. pneumophila* proteins inside DCs is critical for maximal stimulation of CD4^+^ T cells. This indicates that restriction of *L. pneumophila* replication could be important to the ability of DCs to present bacterial antigens to T cells and direct subsequent adaptive immune responses [41]. Interestingly, DCs are able to limit the intracellular replication of several other pathogens that are capable of replicating in macrophages, such as *Listeria monocytogenes*, *Mycobacterium tuberculosis* and *Salmonella enterica* Serovar Typhimurium [42]–[45].

Thus, it appears that there are inherent differences between DCs and macrophages with respect to their abilities to restrict replication of intracellular pathogens. We show here that one of these differences involves the ability of DCs to rapidly activate a cell intrinsic apoptotic cell death pathway in response to the intracellular pathogen *L. pneumophila*.

## Results

### Canonical pathogen surveillance pathways are not required for restriction of *L. pneumophila* replication by DCs

**Figure 1:**
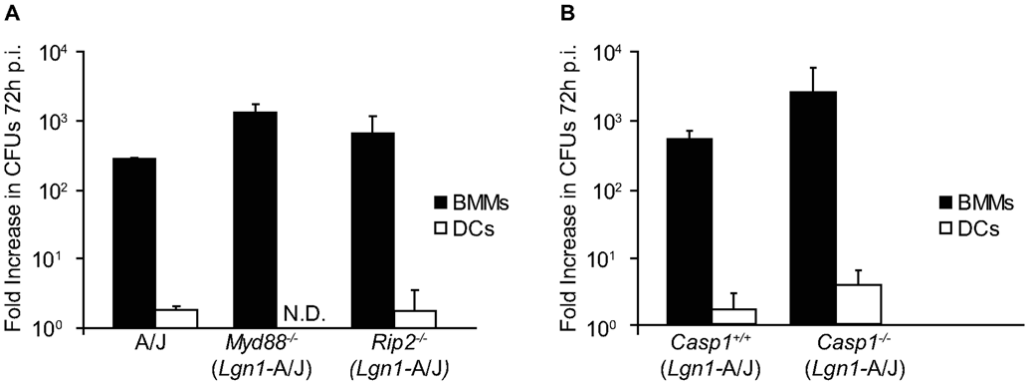
Restriction of *L. pneumophila* replication in DCs does not require signaling by MyD88, Rip2 or caspase-1. (A) BMMs (closed bars) and DCs (open bars) from A/J, *Myd88^−/−^* and *Rip2^−/−^* mice were infected with *L. pneumophila* WT for 72 h. Intracellular replication is determined by dividing *L. pneumophila* CFUs recovered at 72 h by the CFUs recovered at 1 h post infection. (B) BMMs (closed bars) and DCs (open bars) derived from *Casp1^−/−^* or the *Casp1^+/+^* littermate mice were infected with *L. pneumophila* WT. Intracellular replication is determined by dividing *L. pneumophila* CFUs recovered at 72 h by the CFUs recovered at 1 h post infection. Cells were homozygous for the permissive *Lgn1* allele from the A/J mouse as indicated. Data are the mean±SD from three independent wells. N.D. = not detectable.

Signaling through TLRs in macrophages results in enhanced phagocytosis and phagosome fusion with lysosomes [46]. Thus, innate immune recognition of *L. pneumophila* could activate cellular processes that control bacterial replication in DCs. Cells deficient in the adapters MyD88 or Rip2 were used to interfere with the TLR and Nod signaling pathways respectively, to determine whether *L. pneumophila* replication in DCs is restricted by activation of signaling pathways controlled by innate immune receptors. Replication of *L. pneumophila* was not detected in DCs derived from A/J mice, which are defective for Naip5 signaling, or from A/J-derived mice deficient in either MyD88 or Rip2 (Figure 1A). By contrast, exponential replication of *L. pneumophila* occurred in the macrophages derived from these mice (Figure 1A). *L. pneumophila* intracellular replication was not observed in DCs derived from mice deficient in both MyD88 and Trif (data not shown), indicating that the lack of both of these TLR adaptor proteins did not restore *L. pneumophila* intracellular replication in DCs. Thus, DC restriction of *L. pneumophila* replication does not require TLR signaling through MyD88 and Trif or Nod1/2 signaling through Rip2.

Mouse macrophages restrict *L. pneumophila* replication by inducing a cell death pathway controlled by Naip5 and caspase-1 [37]. Mouse macrophages become permissive for *L. pneumophila* replication if they are homozygous for the permissive *Naip5* gene encoded in the A/J mouse or if caspase-1 is absent [35]–[37]. Intracellular replication of *L. pneumophila* was examined in DCs derived from Naip5-deficient mice to determine if the Naip5 protein produced by A/J-derived DCs retained an activity sufficient to restrict replication. The *Naip5* ^−/−^ DCs did not support replication of *L. pneumophila* (Figure S1). It remained possible that proteins other than Naip5 might activate a caspase-1-dependent pathway that prevented *L. pneumophila* replication in DCs from A/J mice. To test this possibility, *L. pneumophila* replication was measured in DCs derived from caspase-1-deficient mice homozygous for the A/J *Naip5* allele (*Casp1^−/−^*). *L. pneumophila* replication was not detected in *Casp1^−/−^* DCs, whereas, *L. pneumophila* replication was similar in BMMs from these same *Casp1^−/−^* and caspase-1-sufficient mice (*Casp1^+/+^*) (Figure 1B). Thus, Naip5 and caspase-1 are not required for DC restriction of *L. pneumophila* replication.

### DC apoptosis occurs rapidly after *L. pneumophila* infection

**Figure 2:**
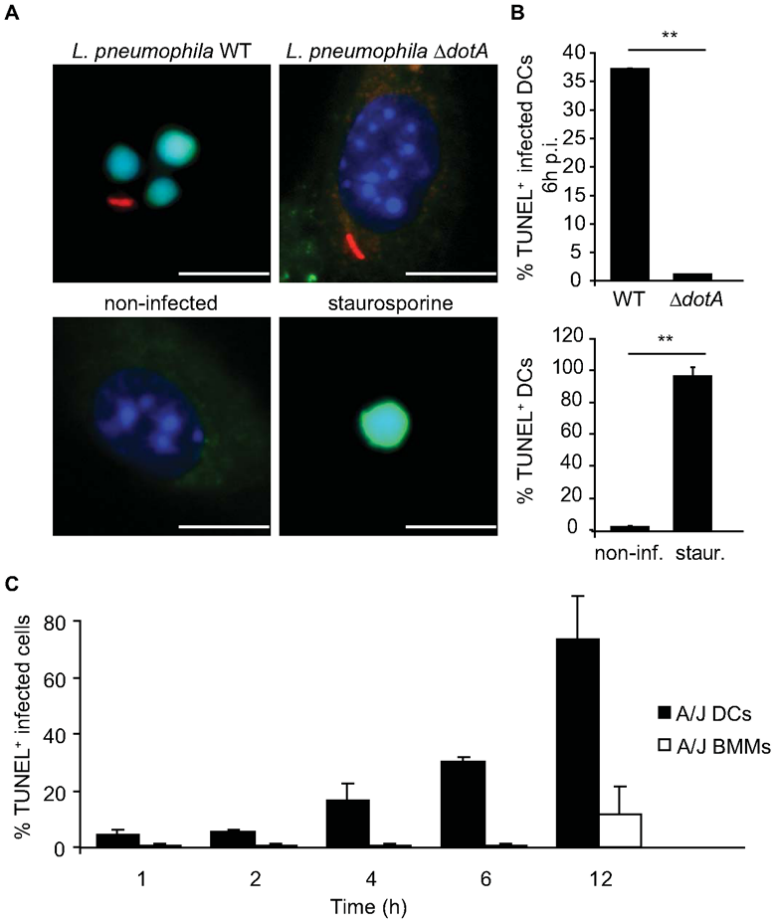
*L. pneumophila* infection of DCs induces nuclear DNA fragmentation. (A) Fluorescence micrographs show TUNEL staining (green) of DCs from A/J mice infected for 6 h with either *L. pneumophila* WT (top left panel) or Δ*dotA* (top right panel). Total DNA was stained with DAPI (blue) and bacteria are red. Non-infected DCs (bottom left panel) or DCs treated with staurosporine for 5 h (bottom right panel) were used as negative and positive controls, respectively. (B) Quantification of the percentage of infected cells that were TUNEL positive (top graph); Quantification of the percentage of total cells that were TUNEL positive in the non-infected DCs and staurosporine-treated DCs (bottom graph). (C) Displayed are the percentage of *L. pneumophila* WT infected DCs (closed bars) or BMMs (open bars) that were TUNEL positive at 1, 2, 4, 6 and 12 h after infection. Data are represented by the mean±SD of 500 cells counted per each coverslip in triplicate. ** p<0.01. Bar = 10 µm.

Although caspase-1-mediated cell death was not required for DCs to restrict the replication of *L. pneumophila*, it remained possible that another cell death pathway could be important for this process. Thus, we examined whether apoptosis occurred upon *L. pneumophila* infection of DCs. TdT-mediated dUTP-biotin nick end-labeling (TUNEL) analysis was performed on DCs infected for 6 hours with either wild type (WT) *L. pneumophila* or the isogenic Δ*dotA* strain that has a nonfunctional Dot/Icm secretion system. Examination of DCs that had internalized WT *L. pneumophila* revealed that 37% were TUNEL positive (Figure 2A and 2B, top panel). Only 1% of DCs containing the Δ*dotA* strain were TUNEL positive (Figure 2A and 2B, top panel). The majority of DCs were TUNEL positive following induction of apoptosis with staurosporine (staur), a broad-spectrum protein kinase inhibitor (Figure 2B, bottom panel). Similar results were obtained using *Casp1^−/−^* DCs (Figure S2), indicating that the absence of caspase-1 did not prevent apoptosis in DCs infected with *L. pneumophila*.

Macrophages and DCs were infected with WT *L. pneumophila* to compare the kinetics of apoptosis. At 1-hour post infection, infected DCs became TUNEL positive, whereas, TUNEL-positive macrophages were not apparent until12-hours post infection (Figure 2C). In addition to using TUNEL staining, the kinetics of apoptosis was determined by measuring caspase-3/7 activity in DCs and macrophages after *L. pneumophila* infection. At 4-hours post infection there was a significant Dot/Icm-dependent increase in caspase-3/7 activity in DC extracts, but not in corresponding macrophage extracts (Figure S3). A significant increase in Caspase-3/7 activity was not observed for macrophages until 11-hours post infection (Figure S3). Thus, apoptosis in DCs was induced by *L. pneumophila* with faster kinetics than in similarly infected macrophages.

### Caspase-3-mediated effector responses are induced by *L. pneumophila* after DC infection

**Figure 3:**
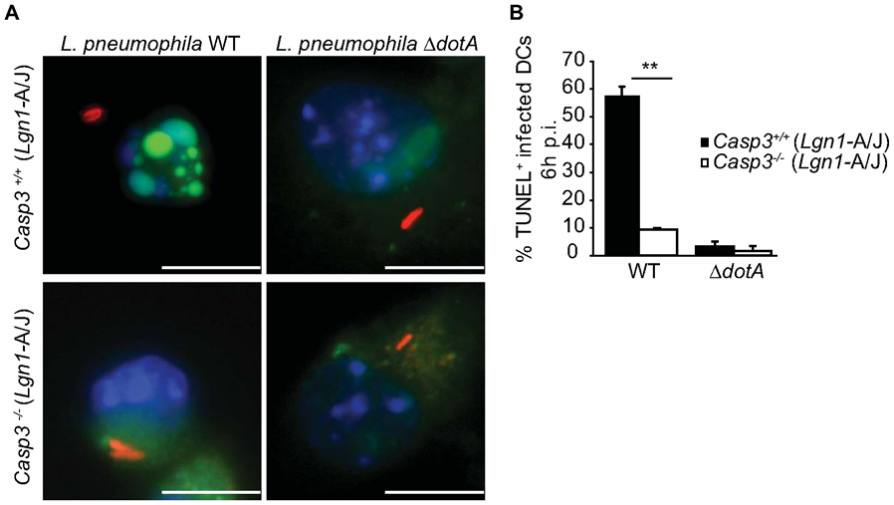
Caspase-3 is required for nuclear DNA fragmentation following *L. pneumophila* infection. (A) Fluorescence micrographs show TUNEL staining (green) of *Casp3^+/+^* and *Casp3^−/−^* DCs infected for 6 h with either *L. pneumophila* WT (left panel) or Δ*dotA* (right panel). Total DNA was stained with DAPI (blue) and bacteria are red. (B) The graph shows percentage of infected *Casp3^+/+^* (closed bars) and *Casp3^−/−^* DCs (open bars) that were TUNEL positive. Cells were homozygous for the permissive *Lgn1* allele from the A/J mouse as indicated. Data are represented by the mean±SD of 500 cells counted per each coverslip in triplicate. ** p<0.01. Bar = 10 µm.

Caspase-3 mediates many of the downstream effector responses in the apoptotic cell death pathway, including fragmentation of DNA in the nucleus [47]. Caspase-3-deficient mice (*Casp3^−/−^*) were used to determine whether DNA fragmentation induced after *L. pneumophila* infection of DCs was due to induction of the apoptotic cell death pathway. TUNEL analysis performed on DCs derived from *Casp3^−/−^* and *Casp3^+/+^* mice 6 hours after infection with *L. pneumophila* revealed that 57% of the infected *Casp3^+/+^* DCs were TUNEL positive, whereas only 9.5% of *Casp3^−/−^* DCs infected with WT *L. pneumophila* were TUNEL positive (Figure 3A, left panel and 3B). Both *Casp3^−/−^* and *Casp3^+/+^* DCs infected with the *ΔdotA* strain showed minimal TUNEL staining (Figure 3A, right panel and 3B). Thus, *L. pneumophila* infection of DCs rapidly activates downstream components of the apoptotic cell death pathway.

### Caspase-3 is involved in DC restriction of *L. pneumophila* replication

**Figure 4:**
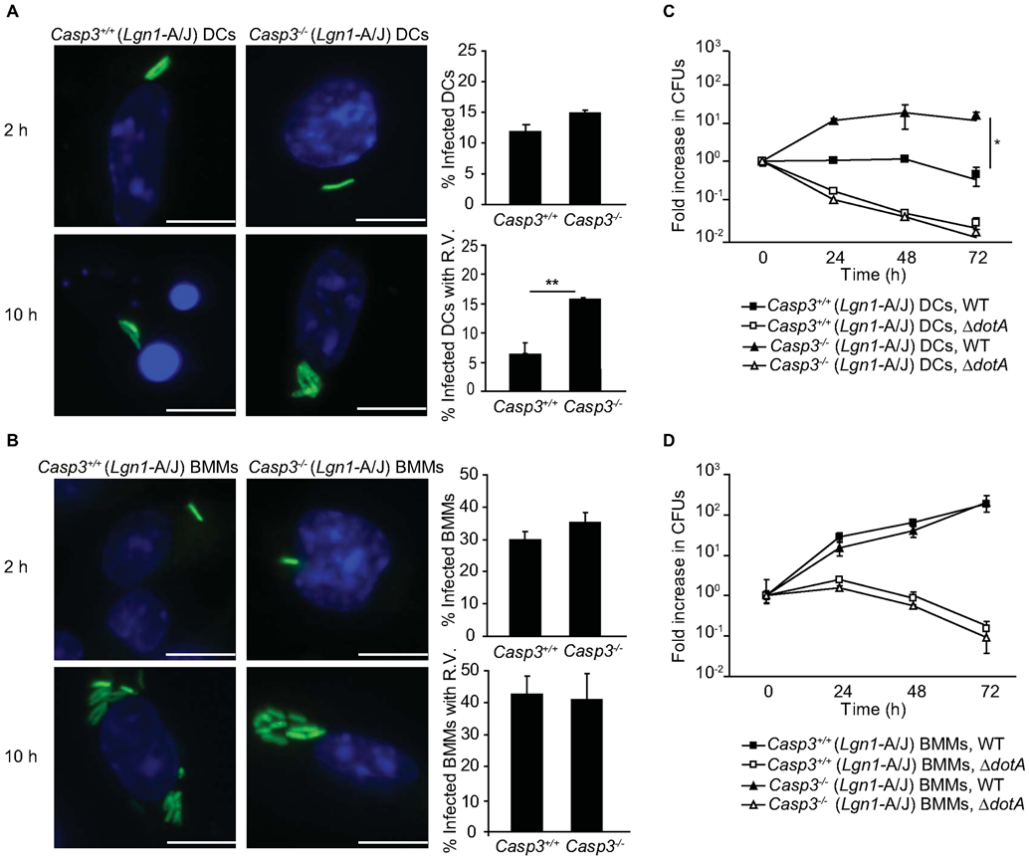
Caspase-3 is required for the restriction of *L. pneumophila* replication in DCs but not macrophages. (A) *Casp3^+/+^* and *Casp3^−/−^* DCs were infected with *L. pneumophila* WT (green) and fixed at either 2 or 10 h after infection. Total DNA was stained with DAPI (blue). On the right are graphical representations of the percentage of infected *Casp3^+/+^* and *Casp3^−/−^* DCs at 2 h post infection and the percentage of infected DCs with vacuoles containing replicating *L. pneumophila* at 10 h post infection. (B) Fluorescence micrographs of *Casp3^+/+^* and *Casp3^−/−^* BMMs (blue) that were fixed at either 2 or 10 h after infection with *L. pneumophila* WT (green); On the right are graphical representations of the percentage of infected BMMs at 2 h post infection and the percentage of infected BMMs with vacuoles containing replicating *L. pneumophila* at 10 h post infection. Data represent the mean±SD of 500 cells counted per coverslip in triplicate. R.V. = vacuoles containing replicating bacteria. ** p<0.01. Bar = 10 µm. (C) DCs or (D) BMMs from *Casp3^+/+^* (triangles) or *Casp3^−/−^* mice (squares) were infected with either *L. pneumophila* WT (closed symbols) or Δ*dotA* (open symbols) and intracellular bacterial replication was measured over a period of 72 h. The fold increase in replication was determined by dividing *L. pneumophila* CFUs recovered at the indicated time point by the *L. pneumophila* CFUs recovered at 1 h post infection. Cells were homozygous for the permissive *Lgn1* allele from the A/J mouse as indicated. Data represent the mean±SD from three independent wells. * p<0.05.

To determine whether activation of the apoptotic cell death pathway was important for DC restriction of *L. pneumophila* replication, DCs from A/J-derived *Casp3^−/−^* and *Casp3^+/+^* mice infected with WT *L. pneumophila* were examined by fluorescence microscopy. The efficiency of *L. pneumophila* internalization determined 2 hours after infection was equivalent for *Casp3^−/−^* and *Casp3^+/+^* DCs (Figure 4A, top panel). When DCs were examined 10 hours after infection, there was a significant increase in the percentage of infected *Casp3^−/−^* DCs that contained vacuoles supporting *L. pneumophila* replication (R.V.) (19%) compared to *Casp3^+/+^* DCs (6%) (Figure 4A, bottom panel). Representative images in Figure 4A show that the number of *L. pneumophila* in vacuoles that supported replication was higher in *Casp3^−/−^* DCs, and that most of the infected *Casp3^+/+^* DCs had condensed or fragmented nuclei. These data were corroborated by determining colony-forming units (CFUs) over time. There was roughly a 10-fold increase in *L. pneumophila* CFUs 72 hours after *Casp3* ^−/−^ DCs were infected with WT *L. pneumophila* compared to a slight decrease in CFUs recovered from *Casp3^+/+^* DCs at 72 hours (Figure 4C). DCs eliminated the Δ*dotA* strain with equal efficiency. Macrophages derived from these mice were infected in parallel. The infected *Casp3^+/+^* macrophages had normal nuclei (Figure 4B) and supported *L. pneumophila* replication to similar levels as the *Casp3^−/−^* macrophages (Figure 4B and 4D). These data indicate that caspase-3 plays a role in restricting *L. pneumophila* replication in DCs, but not macrophages.

### Cell death mediated by Bax and Bak restricts *L. pneumophila* replication in DCs

**Figure 5:**
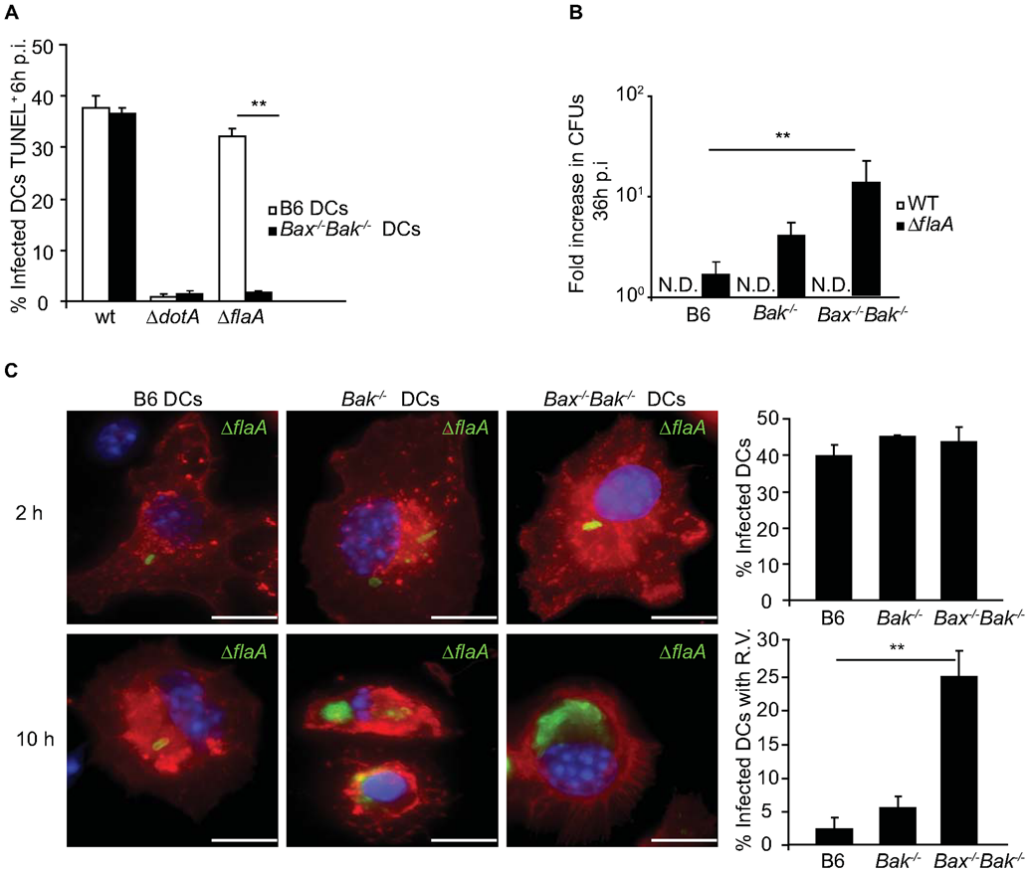
*Bax* and *Bak* are required for *L. pneumophila* growth restriction in DCs. (A) The graph shows percentage of B6 (open bars) and *Bax^−/−^Bak^−/−^* DCs (closed bars) infected with *L. pneumophila* WT, Δ*dotA* or Δ*flaA* that were TUNEL positive at 6 h post infection. Data represent the mean±SD of 300 cells counted per coverslip in triplicate. ** p<0.01. (B) B6, *Bak^−/−^* and Bax^−/−^Bak^−/−^ were infected with either *L. pneumophila* WT (white bars) or Δ*flaA* (black bars) for 36 h. Intracellular replication was determined by dividing *L. pneumophila* CFUs recovered at 36 h by the CFUs recovered at 1 h post infection. Data for each time point are the average of values obtained from three independent wells. ** p<0.01. (C) Fluorescence micrographs of B6, *Bak^−/−^* and *Bax^−/−^Bak^−/−^* DCs that were infected with *L. pneumophila* Δ*flaA* and fixed at either 2 h or 10 h post infection. DCs were stained with an antibody specific for MHC II (red), DAPI (blue) and an anti-*L. pneumophila* antibody (green). On the right are graphical representations of the percentage of B6, *Bak^−/−^* and *Bax^−/−^Bak^−/−^* DCs infected at 2 h and the percentage of infected B6; *Bak^−/−^* and *Bax^−/−^Bak^−/−^* DCs with vacuoles containing replicating bacteria at 10 h post infection. Data represent the mean±SD of 500 cells counted per coverslip in triplicate. All cells had a dominant *Lgn1* allele producing a functional Naip5 protein. R.V. = vacuoles containing replicating bacteria. N.D. = not detectable. ** p<0.01. Bar = 10 µm.

Bax and Bak play a central role in regulating apoptosis. When activated by members of the BH3-only protein family, Bax and Bak create a channel in the membrane of mitochondria that releases cytochrome c. This results in activation of the apoptosome and the subsequent activation of effector caspases, such as caspase-3 [48]–[51]. DCs derived from C57BL/6 (B6) and from mice deficient in Bak (*Bak^−/−^*) or both Bax and Bak (*Bax^−/−^Bak^−/−^*) were analyzed to determine if Bax and Bak have a role in cell death induced by *L. pneumophila*. TUNEL analysis demonstrated that WT *L. pneumophila* induced equivalent levels of cell death in DCs derived from B6 and *Bax^−/−^Bak^−/−^* mice (Figure 5A), suggesting that the Naip5-dependent pathway of cell death remained functional in DCs. A *L. pneumophila* strain containing an in-frame deletion of the *flaA* gene encoding flagellin was used to bypass Naip5-mediated cell death [38]–[40]. A dramatic reduction in cell death was observed for *Bax^−/−^Bak^−/−^* DCs infected with *L. pneumophila ΔflaA* (Figure 5A). Measurements of caspase-3/7 activity following infection of DCs confirmed that Bax and Bak were required for induction of apoptosis by *L. pneumophila ΔflaA* (Table 1). Thus, *L. pneumophila* independently induces DC cell death by a Bax/Bak-dependent pathway and a Naip5-dependent pathway.

**Table 1:** Caspase-3/7 activity 6 h post-infection in relative fluorescence units.

*L. pneumophila*	B6 DCs±SD	*Bak^−/−^* DCs±SD	*Bax^−/−^Bak^−/−^* DCs±SD
non-infected	19133±1950	13433±680	10000±624
*ΔflaA*	32467±1358	12333±404	8567±321
*ΔdotA*	17633±1357	10666±404	8433±1001

Replication of WT *L. pneumophila* was not detected in either *Bak^−/−^* or *Bax^−/−^Bak^−/−^* DCs (Figure 5B), which is consistent with the Naip5-mediated pathway being operational in these cells. *L. pneumophila ΔflaA* replicated to similar levels as WT *L. pneumophila* in DCs derived from *Casp3* ^−/−^ mice homozygous for the A/J *Naip5* allele (Figure S4), indicating that eliminating flagellin does not significantly enhance the capacity of *L. pneumophila* to replicate in DCs with a genetic defect in the Naip5 signaling pathway. DCs from *Bax^−/−^Bak^−/−^* mice supported replication of *L. pneumophila ΔflaA*, whereas, replication of *L. pneumophila ΔflaA* was not detected in DCs from control B6 mice (Figure 5B). Limited replication of the Δ*flaA* strain was observed in *Bak^−/−^* DCs; however, replication was not as robust as that observed in the *Bax^−/−^Bak^−/−^* DCs (Figure 5B). Single cell analysis revealed that the efficiency of infection was equivalent in B6, *Bak^−/−^* and *Bax^−/−^Bak^−/−^* DCs (Figure 5C, top panel). Large vacuoles harboring replicating bacteria were abundant in *Bax^−/−^Bak^−/−^* DCs infected for 10-hours with *L. pneumophila ΔflaA* (Figure 5C, bottom panel), whereas, vacuoles containing replicating *L. pneumophila ΔflaA* were rare in the B6 and *Bak^−/−^* DCs.

**Figure 6:**
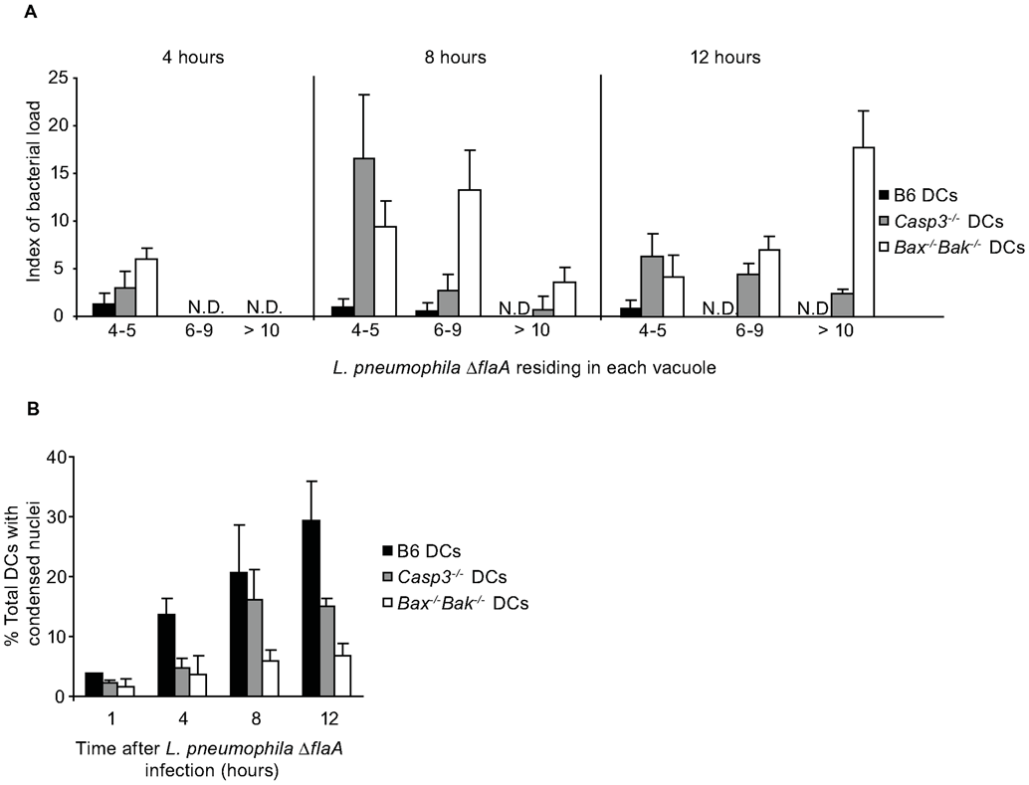
Enhanced replication of *L. pneumophila* in DCs correlates with reduced apoptosis. (A) The graph shows the percentage of infected B6 (black bars), *Casp3^−/−^* (gray bars) or *Bax^−/−^Bak^−/−^* DCs (white bars) that form vacuoles containing 4–5, 6–9 or >10 replicating *L. pneumophila* Δ*flaA* at 4, 8 and 12 h post infection. The index of bacterial load was calculated by dividing the percentage of vacuoles containing the indicated number of bacteria at the time points given by the percentage of infected DCs at 2 h p.i. and multiplying this number by 100. (B) The graph shows the percentage of total B6 (black bars), *Casp3^−/−^* (gray bars) and *Bax^−/−^Bak^−/−^* DCs (white bars) that had condensed nuclei at 1, 4, 8 and 12 hours after *L. pneumophila* Δ*flaA* infection. All cells had a dominant *Lgn1* allele producing a functional Naip5 protein. Data represent the mean±SD of 300 cells counted per coverslip in triplicate.

The development of vacuoles containing replicating *L. pneumophila ΔflaA* was evaluated in DCs derived from B6, *Casp3* ^−/−^ and *Bax^−/−^Bak^−/−^* mice. Vacuoles containing replicating *L. pneumophila ΔflaA* were detectable in both *Casp3* ^−/−^, and *Bax^−/−^Bak^−/−^* DCs at 8-hours post infection (Figure 6A). Large vacuoles containing >10 *L. pneumophila ΔflaA* were frequent in the *Bax^−/−^Bak^−/−^* DCs at 12-hours post infection, but were found less frequently in the *Casp3* ^−/−^ DCs (Figure 6A). Although *Casp3* ^−/−^ DCs exhibited enhanced resistance to cell death induced by *L. pneumophila ΔflaA*, they were not as resistant to cell death as the *Bax^−/−^Bak^−/−^* DCs (Figure 6B), which likely explains why the *Bax^−/−^Bak^−/−^* DCs were slightly more permissive for replication of *L. pneumophila ΔflaA* at 12-hours post infection compared to the *Casp3* ^−/−^ DCs. These data indicate *L. pneumophila* activation of the intrinsic cell death pathway in DCs is sufficient to limit intracellular replication.

### Bcl-2 overproduction antagonizes restriction of *L. pneumophila* replication by DCs

**Figure 7:**
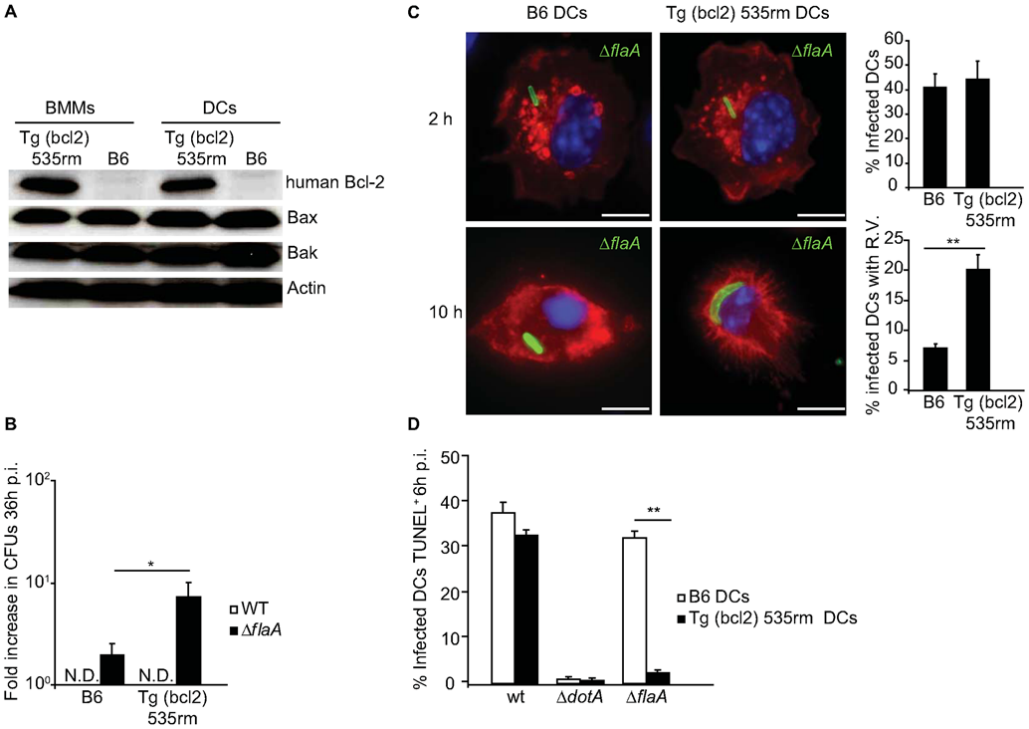
*L. pneumophila* replication occurs in DCs overexpressing Bcl-2. (A) Immunoblot analysis of total human Bcl-2, Bax and Bak expression in Tg (bcl2) 535rm BMMs and DCs or the respective B6 littermates. Blots were reprobed for actin as a loading control. (B) B6 and Tg (bcl2) 535rm DCs were infected with either *L. pneumophila* WT (white bars) or Δ*flaA* (black bars) for 36 h. Intracellular replication was determined by dividing the CFUs recovered at 36 h by the CFUs recovered at 1 h after infection. Data for each time point are the average of values obtained from three independent wells. * p<0.05. N.D. = not detectable. (C) Fluorescence micrographs of B6 or Tg (bcl2) 535rm DCs that were infected with *L. pneumophila* Δ*flaA* and fixed at either 2 h or 10 h post infection. DCs were stained with an antibody specific for MHC II (red), DAPI (blue) and an anti-*L. pneumophila* antibody (green). On the right are graphical representations of the percentage of infected B6 or Tg (bcl2) 535rm DCs at 2 h post infection and the percentage of infected DCs with vacuoles containing replicating *L. pneumophila* Δ*flaA* at 10 h post infection. Data represent the mean±SD representative of 500 cells counted per coverslip in triplicate. R.V. = vacuoles containing replicating bacteria. ** p<0.01. Bar = 10 µm. (D) The graph shows the percentage of B6 (open bars) and Tg (bcl2) 535rm DCs (closed bars) infected with *L. pneumophila* WT, Δ*dotA* or Δ*flaA* that were TUNEL positive at 6 h post infection. All cells had a dominant *Lgn1* allele producing a functional Naip5 protein. Data are represented by the mean±SD of 300 cells per coverslip in triplicate. ** p<0.01.

Bcl-2 is a pro-survival protein that regulates apoptosis [52],[53]. Overexpression of pro-survival proteins such as those from the Bcl-2 family can block mitochondria membrane permeabilization and prevent apoptosis [54]–[56]. DCs from transgenic mice expressing human *BCL2* under the control of the CD68 promoter (Tg(bcl2) 535rm) (Jamieson & Medhzitov, unpublished data) were used to determine whether overproduction of Bcl-2 could interfere with the ability of DCs to restrict *L. pneumophila* replication. Immunoblot analysis confirmed that both macrophages and DCs derived from Tg(bcl2) 535rm mice produced human Bcl-2, and that overproduction of Bcl-2 did not affect the levels of Bax and Bak in these cells (Figure 7A). Replication of WT *L. pneumophila* was not observed in Tg(bcl2) 535rm DCs, presumably because these cells produce a functional Naip5 protein (Figure 7B). Replication of the Δ*flaA* strain was observed in the Tg(bcl2) 535rm DCs, but not in control DCs from B6 mice (Figure 7B). Single cell analysis confirmed replication of the Δ*flaA* strain in Tg(bcl2) 535rm DCs (Figure 7C). At 10-hours post infection, 21% of the Δ*flaA*-infected Tg(bcl2) 535rm DCs had large vacuoles containing replicating *L. pneumophila*, and most of the infected Tg(bcl2) 535rm DCs were devoid of apoptotic features, such as condensed and fragmented nuclei, that were observed in infected control DCs derived from B6 mice (Figure 7C). TUNEL staining confirmed that the Tg(bcl2) 535rm DCs were more resistant to apoptosis after infection by *L. pneumophila* Δ*flaA* compared to control B6 DCs (Figure 7D). Thus, Bcl-2 overproduction limited DC apoptosis in response to *L. pneumophila* and resulted in enhanced intracellular replication.

### DCs have a unique ability to efficiently restrict *L. pneumophila* replication by apoptosis

**Figure 8:**
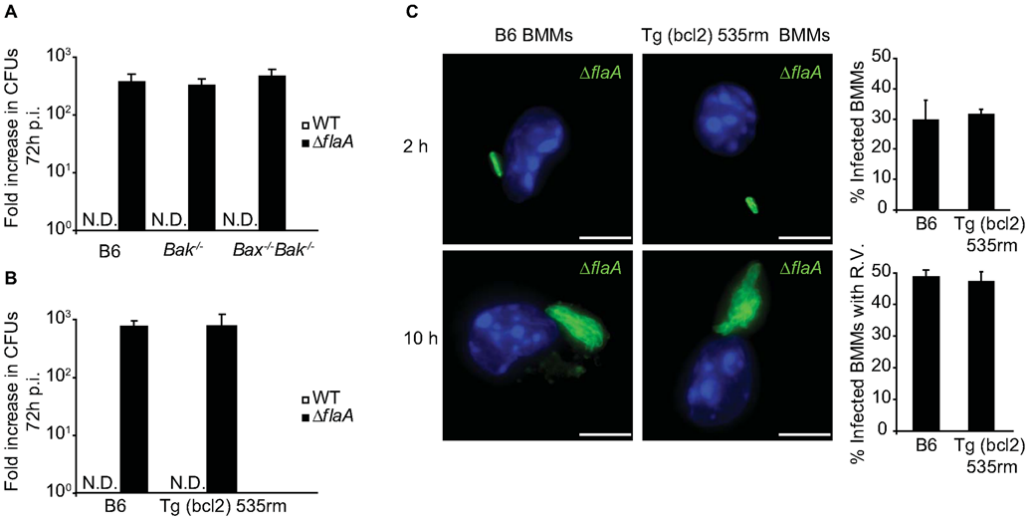
Interfering with Bax and Bak function does not enhance *L. pneumophila* replication in macrophages. (A) B6, *Bak^−/−^* and *Bax^−/−^Bak^−/−^* BMMs or (B) B6 and Tg (bcl2) 535rm BMMs were infected with either *L. pneumophila* WT (white bars) or *ΔflaA* (black bars) for 72 hours. Intracellular replication is determined by dividing the *L. pneumophila* CFUs recovered at 72 h by the CFUs recovered 1 h after infection. Data are the average of values obtained from three independent wells. N.D. = not detectable. (C) Fluorescence micrographs of B6 and Tg (bcl2) 535rm BMMs that were infected with *L. pneumophila ΔflaA* and fixed either at 2 h or 10 h post infection. BMMs were stained with DAPI (blue) and an anti-*L. pneumophila* antibody (green). On the right are graphical representations of the percentage of B6 or Tg (bcl2) 535rm BMMs infected at 2 h and the percentage of infected BMMs with vacuoles containing replicating *L. pneumophila* at 10 h post infection. Data represent the mean±SD of 500 cells counted per coverslip in triplicate. All cells had a dominant *Lgn1* allele producing a functional Naip5 protein. R.V. = vacuoles containing replicating bacteria. Bar = 10 µm.

Macrophages derived from *Bax^−/−^Bak^−/−^* and Tg(bcl2) 535rm mice were used to determine whether rapid induction of programmed cell death as a mechanism to restrict *L. pneumophila* replication was an exclusive property displayed by DCs. Replication of WT *L. pneumophila* was restricted by the *Bax^−/−^Bak^−/−^* macrophages and Tg(bcl2) 535rm macrophages as efficiently as control B6 macrophages (Figure 8A and 8B). When the Δ*flaA* strain was used to bypass Naip5-mediated growth restriction, bacterial replication was not enhanced in the *Bax^−/−^Bak^−/−^* macrophages or Tg(bcl2) 535rm macrophages compared to control B6 macrophages (Figure 8A and 8B). Single cell analysis confirmed these growth curve results, and showed that Bax and Bak function was not required for Naip5-mediated growth restriction of WT *L. pneumophila* and had no measurable effect on limiting the growth of the Δ*flaA* strain in macrophages (Figure 8C).

**Figure 9:**
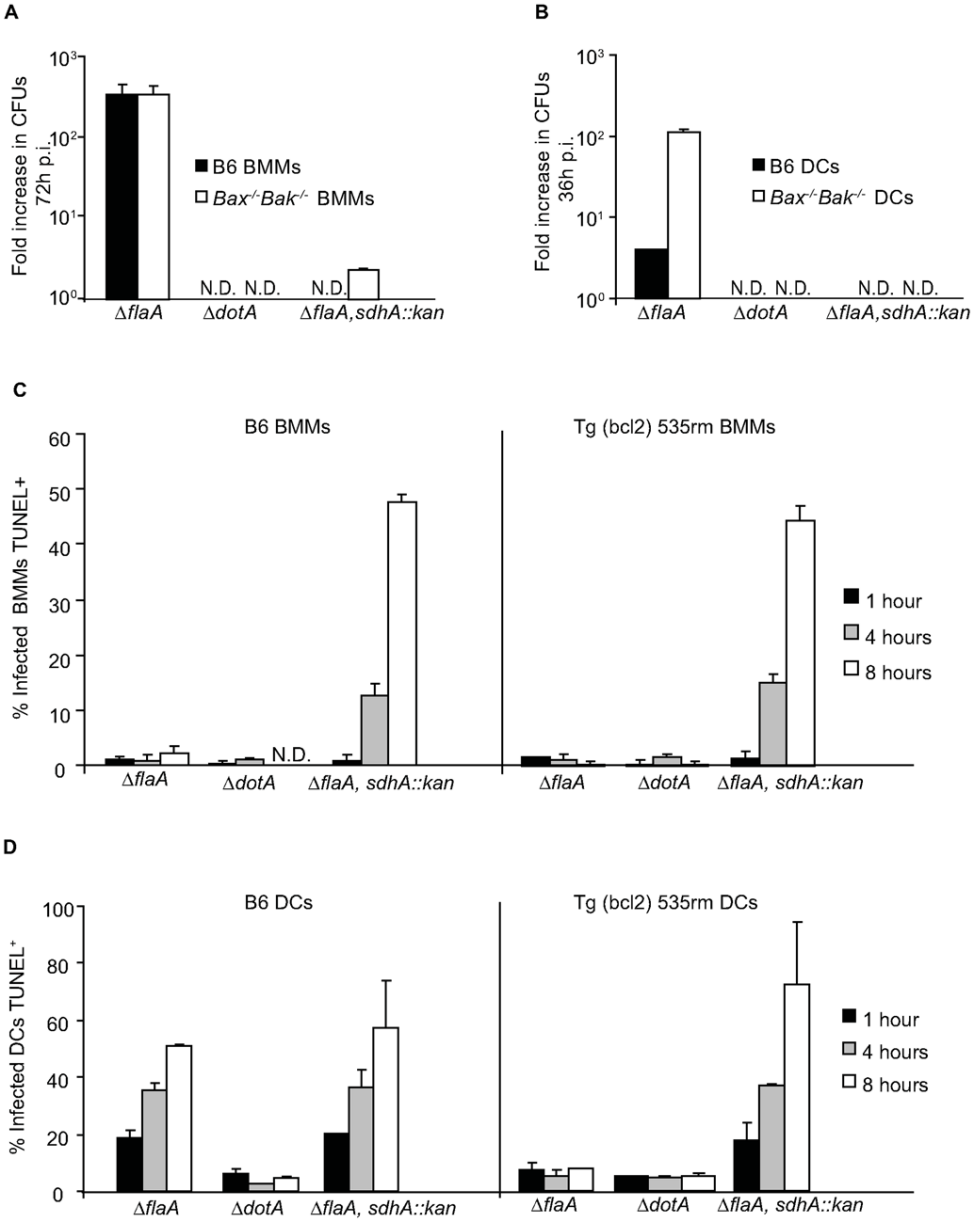
*L. pneumophila sdhA* mutants induce rapid cell death in macrophages and DCs by a pathway that does not require Bax and Bak. (A) Intracellular replication of *L. pneumophila* Δ*flaA*, Δ*dotA* and Δ*flaA*, *sdhA::kan* was measured in B6 (black bars) and *Bax^−/−^Bak^−/−^* BMMs (white bars) at 72 h after infection. The fold increase in intracellular replication was determined by dividing *L. pneumophila* CFUs recovered at 72 h by the CFUs recovered at 1 h post infection. (B) Intracellular replication of *L. pneumophila* Δ*flaA*, Δ*dotA* and Δ*flaA*, *sdhA::kan* in B6 DCs (black bars) and *Bax^−/−^Bak^−/−^* DCs (white bars) at 36 h after infection. The fold increase in intracellular replication was determined by dividing *L. pneumophila* CFUs recovered at 36 h by the *L. pneumophila* CFUs recovered at 1 h post infection. Data are the mean±SD from three independent wells. N.D. = not detectable. (C) The graph shows the percentage of B6 and Tg (bcl2) 535rm BMMs or (D) DCs infected with *L. pneumophila* Δ*flaA*, Δ*dotA* or Δ*flaA*, *sdhA::kan* that were TUNEL positive at 1 h (black bars), 4 h (gray bars) and 8 h (white bars) after infection. All cells had a dominant *Lgn1* allele producing a functional Naip5 protein. Data are represented by the mean±SD of 300 cells counted per each coverslip in triplicate.

Previous studies have shown that macrophages infected with a *L. pneumophila* mutant deficient in the effector protein SdhA undergo rapid cell death by an unknown pathway [57]. This observation suggests that one possible reason DCs die quickly after *L. pneumophila* infection is because a proposed anti-apoptotic activity mediated by the translocated SdhA protein might not be effective at preventing cell death in DCs. This would explain why the phenotype of DCs infected by *L. pneumophila* capable of translocating the SdhA protein appears to be similar to the phenotype of macrophages infected by an *sdhA* mutant. If this hypothesis is correct, then perturbing cell death pathways activated by Bax and Bak should restore replication of an *sdhA* mutant in macrophages, and the elimination of SdhA should not affect replication of *L. pneumophila* in DCs deficient in Bax and Bak signaling. To test this hypothesis we inactivated *sdhA* in the *L. pneumophila ΔflaA* strain to generate *L. pneumophila* Δ*flaA*, *sdhA*::*kan*. Elimination of Bax and Bak did not restore replication of *L. pneumophila* Δ*flaA*, *sdhA::kan* in macrophages (Figure 9A) and the *L. pneumophila* Δ*flaA*, *sdhA::kan* strain was unable to replicate in *Bax^−/−^Bak^−/−^* DCs (Figure 9B). After infection by *L. pneumophila* Δ*flaA*, *sdhA*::*kan*, cell death levels measured by TUNEL staining were similar in Tg(bcl2) 535rm macrophages and control B6 macrophages (Figure 9C). The *L. pneumophila* Δ*flaA*, *sdhA*::*kan* strain also induced cell death in DCs derived from Tg(bcl2) 535rm mice (Figure 9D). Thus, the *L. pneumophila sdhA* mutant phenotype was similar in both macrophages and DCs, which indicates that SdhA is necessary to prevent *L. pneumophila* from killing both macrophages and DCs by a pathway that does not require Bax and Bak function.

## Discussion

Two cell death pathways were found to restrict *L. pneumophila* replication in DCs. The first pathway was described previously in macrophages and involved activation of Naip5 by a process requiring *L. pneumophila* flagellin [37]–[40]. It had been shown clearly that stimulation of Naip5 by *L. pneumophila* flagellin results in the activation of caspase-1 [37]–[40], which is a critical mediator of pyroptosis. Recent data indicate that Naip5 activation of caspase-1 also results in the activation of caspase-7 [58], and that Naip5-dependent activation of caspase-7 is important for restriction of *L. pneumophila* replication in mouse macrophages. Many details of the Naip5 signaling pathway remain to be determined, including the full repertoire of proteins required for Naip5-mediated cell death and all the cell types capable of restricting the replication of *L. pneumophila* by this pathway. Our data help to answer some of these questions by showing that components of the Naip5 pathway required for flagellin sensing and downstream effector responses are functioning in DCs. Additionally, the observation that overproduction of Bcl-2 or elimination of Bax and Bak did not affect restriction of WT *L. pneumophila* replication in macrophages and DCs with a functional Naip5 protein provides evidence that this pathway is not functionally dependent on the mitochondrial pathway of apoptosis. Thus, both macrophages and DCs have the capacity to undergo Naip5-dependent pyroptosis. In addition to restricting pathogen replication, activation of caspase-1 during this response generates bioactive IL-1β and IL-18 to stimulate additional antimicrobial responses and promote the recruitment of other immune cells [59]–[62]. This suggests that pyroptosis is a general innate immune response mediated by both macrophages and DCs to initiate early pro-inflammatory events at the site of microbial infection.

A second cell death pathway, which involved Bax and Bak regulation of caspase-3 activation, was found to efficiently restrict *L. pneumophila* replication in DCs. When the pyroptosis pathway was inactivated, either by using DCs with a defective *Naip5* allele or by using *L. pneumophila* that had the gene encoding flagellin deleted, the cell death pathway regulated by Bax and Bak was as efficient as the pyroptosis pathway at restricting replication of *L. pneumophila*. A similar number of replicating *L. pneumophila* were contained in vacuoles in DCs deficient in Bax and Bak at 10-hours post infection (Figure 5C) when compared to macrophages (Figure 8C). Additionally, the number of *L. pneumophila* recovered from DCs deficient in caspase-3 was similar after 24-hours of infection when compared to macrophages (Figure 4). Because the addition of bacteria stimulates the maturation of DCs in culture, and mature DCs become non-phagocytic, *L. pneumophila* replication in cultured DCs was not amplified by reinfection. This explains why replication subsided after *L. pneumophila* exited infected DCs at 24-hours post infection, but continued over a 72-hour period in macrophages (Figure 4). Thus, rapid activation of the intrinsic cell death pathway appears to be the primary mechanism by which DCs from permissive strains of mice restrict the intracellular replication of *L. pneumophila*.

*L. pneumophila* was capable of replication in DCs deficient in caspase-3; however, DCs deficient in both Bax and Bak were more permissive. This suggests that deletion of Bax and Bak more acutely blocks the apoptotic pathway, perhaps because other effector caspases can compensate for caspase-3 deficiency. Consistent with this explanation, *Bax^−/−^Bak^−/−^* mice have severe developmental defects and most die perinatally, whereas, *Casp3^−/−^* mice are viable and have fewer developmental defects [63]–[65]. Accordingly, *L. pneumophila* infection induced the mitochondrial pathway of apoptosis in *Casp3^−/−^* DCs, but the absence of caspase-3 was sufficient to delay cell death for a long enough period of time that vacuoles containing replicating *L. pneumophila* were detected. By contrast, apoptosis was not induced upon *L. pneumophila* infection of *Bax^−/−^Bak^−/−^* DCs and in the absence of cell death *L. pneumophila* was able to replicate for a longer period of time as was indicated by an increase in the number of large vacuoles containing over 10 bacteria. These data also suggest that cell death, as opposed to another activity mediated specifically by caspase-3, was sufficient to restrict *L. pneumophila* replication.

The finding that overproduction of Bcl-2 resulted in enhanced bacterial replication in DCs supports the hypothesis that the mitochondrial pathway of apoptosis is important for restriction of *L. pneumophila* replication in DCs. Bcl-2 functions as a negative regulator of Bax and Bak function, preventing their activation and insertion into the mitochondrial membrane [66],[67]. Thus, the observation that Bcl-2 overproduction phenocopies a deficiency in Bax and Bak indicates that *L. pneumophila* infection of DCs triggers a cell-autonomous response that activates the mitochondrial pathway of apoptosis, leading to restriction of intracellular bacterial proliferation.

Previous studies in macrophages and macrophage-like cells have demonstrated that *L. pneumophila* is capable of activating the mitochondrial pathway of apoptosis [68]–[71]; however, our data indicate that the timing of this response is different in DCs compared to macrophages. In macrophages the response is slower, and morphological signs of apoptosis were typically not observed in cells until the late stages of infection after robust bacterial replication had occurred. Host cell apoptosis induced by *L. pneumophila* in both macrophages and DCs required a functional Dot/Icm secretion system, but not bacterial replication. This suggests that apoptosis is activated in response to either direct activities of bacterial effector proteins translocated by the Dot/Icm system or by host cell disturbances that are caused by the cumulative actions of multiple effector proteins.

The balance of pro-apoptotic to anti-apoptotic factors is important in the regulation of the mitochondrial pathway of apoptosis. Microbial infection affects this balance both by triggering the activation of pro-apoptotic factors and by inducing expression of anti-apoptotic proteins [49], [72]–[74]. For many non-pathogenic bacteria, these two events are balanced and apoptosis is prevented. The added stress on cells infected with pathogenic microbes, however, will typically result in apoptosis unless the pathogen has the ability to alter the function of proteins involved in regulating cell death [75],[76]. Thus, differences in the expression of Bcl-2 family members or in the functioning of effector proteins could account for the faster kinetics of apoptosis in DCs compared to macrophages following *L. pneumophila* infection.

Two effector proteins translocated into host cells by the *L. pneumophila* Dot/Icm system have been implicated in preventing cell death. The effector protein SidF appears to interfere with the function of pro-apoptotic Bcl-2 family members BNIP3 and Bcl-Rambo [77]. Although macrophages infected with a *sidF* mutant show increased apoptosis 14-hours after infection, this increase in apoptosis does not impact bacterial replication greatly [77]. By contrast, the effector SdhA is required to prevent macrophage cell death during infection by a mechanism that is not understood, and the cell death induced by an *sdhA* mutant greatly reduces bacterial replication in macrophages [57]. We found that the *sdhA* mutant induced cell death in both macrophages and DCs, and that this cell death pathway was not inhibited by Bcl-2 over-expression or elimination of Bax and Bak. Additionally, intracellular growth of the *sdhA* mutant was not restored in macrophages deficient in caspase-3 (data not shown). Thus, both macrophages and DCs are equally susceptible to cell death induced by the *sdhA* mutant, and the cell death pathway triggered by the *sdhA* mutant does not require several of the central components of the apoptosis pathway. These data are consistent with there being an intrinsic difference between macrophages and DCs with respect to their ability to activate the mitochondrial cell death pathway in response to *L. pneumophila*.

In addition to *L. pneumophila*, there are many other reports demonstrating that DCs are able to restrict the replication of pathogens capable of growing within macrophages [42]–[45]. DCs are very proficient at migrating from peripheral tissues to the host lymphatic system following exposure to maturation stimuli, such as encounters with microbes. Because of this property, it has been suggested that DCs can function as a “Trojan Horse” capable of systemic dissemination of pathogens internalized at peripheral sites of infection [42],[44],[78]. Here we show that rapid cell death is one mechanism DCs use to avoid being subverted by an intracellular pathogen. In addition to preventing pathogen replication and dissemination, apoptotic DCs harboring intracellular pathogens would become substrates for phagocytosis by neighboring DCs and macrophages, and most mechanisms used by intracellular pathogens to subvert host cellular function would be ineffective as long as the pathogen were residing in an apoptotic cell. Thus, apoptotic bodies containing pathogens would be degraded in lysosomes, resulting in the release of pathogen-derived molecules that could stimulate innate immune receptors and trigger adaptive responses by being presented on the cell surface in association with host MHC proteins. Based on these data, we hypothesize that rapid pathogen-induced apoptosis by DCs is an important innate immune response to intracellular pathogens.

## Materials and Methods

### Bacterial cultures

*L. pneumophila* serogroup 1 strain, Lp01 [18], an isogenic *dotA* mutant strain (Δ*dotA*), and a flagellin-deficient mutant strain (*ΔflaA*) [79] were cultured on charcoal yeast extract agar (CYE) [80] for 2 days prior to use in experiments. The Δ*flaA*, *sdhA*::*kan* strain was cultured on CYE with 10 µg/mL kanamycin. The plasmid pAM239 was used to produce DSred or GFP in the *L. pneumophila* strains indicated [81]. For experiments utilizing bacteria expressing DSred or GFP, *L. pneumophila* was grown on plates supplemented with chloramphenicol (6.25 µg/ml), and DSred or GFP expression was induced after infection by adding IPTG (0.2 mM) to the tissue culture medium.

### Mice

A/J and C57BL/6 (B6) mice were purchased from Jackson Laboratories. *Caspase-1^−/−^* (*Casp1^−/−^*), *Caspase-3^−/−^* (*Casp3^−/−^*), *Myd88^−/−^*, *Rip2* ^−/−^ (*Ripk2^−/−^*;*Rick^−/−^*), *Bak^−/−^*, *Bax^−/−^Bak^−/−^* and *Naip5^−/−^* mice have been described [59], [63], [65], . *Myd88^−/−^Trif^−/−^* mice homozygous for the B6 *Lgn1* allele were provided by R. Medzhitov. *Myd88^−/−^* and *Rip2* ^−/−^ mice were crossed with A/J mice to generate progeny homozygous for the A/J *Lgn1* allele as described previously [31]. *Casp1^−/−^* and *Casp3^−/−^* mice homozygous for the permissive A/J *Lgn1* allele were backcrossed to the A/J background for 4 and 5 generations respectively. Transgenic C57BL/6 mice over expressing human BCL2 under the control of the CD68 promoter (Tg(bcl2) 535rm) (Jamieson & Medhzitov, unpublished data), were kindly provided by R. Medzhitov. All animals were maintained in accordance with the guidelines of the Yale Institutional Animal Use and Care Committee.

### Macrophage and dendritic cell cultures

Bone-marrow derived macrophages (BMMs) were prepared as described previously with some modifications [85]. Briefly, bone marrow was collected from the femurs and tibiae of mice. Cells were plated on non-tissue culture-treated dishes and incubated at 37°C in RPMI-1640 containing 20% heat-inactivated fetal bovine serum (FBS), 30% macrophage colony-stimulating factor (M-CSF)-conditioned medium, and 1% penicillin-streptomycin. On day 7, cells were harvested and resuspended in RPMI 1640 containing 10% FBS and 15% M-CSF-conditioned medium. Cells were then plated in 24-well tissue culture-treated plates and incubated at 37°C. Bone marrow derived-DCs (BMDCs) were prepared as described in Lutz *et al.* [86]. Modifications were as follows. Cells were plated on non-tissue culture-treated dishes and incubated at 37°C in RPMI-1640 supplemented with 10% heat-inactivated FBS, 50 µM 2-mercaptoethanol, 1% penincillin-streptomycin and 1% GM-CSF (DC medium). Cells were harvested and used on day 10.

### Intracellular replication assays

Intracellular replication of *L. pneumophila* in BMMs was measured as described previously [79] and modified slightly for DCs. *L. pneumophila* was added to DCs at a multiplicity of infection (MOI) of 20. The plates were centrifuged at 150 g for 5 minutes (min) and then incubated at 37°C for 30 min. Cells were removed from the wells and DCs were positively selected on magnetic columns using anti-CD11c-coated magnetic beads (Miltenyi Biotech). To remove extracellular bacteria, DCs were washed 3× with PBS containing 2 mM EDTA and 0.5% BSA while bound to the column. DCs were eluted and 2×10^5^ DCs were added to individual wells in 48-well plates. Adherent and non-adherent DCs were taken from individual wells and lysed with sterile H_2_O at the indicated times after infection, and these fractions were pooled with the culture supernatants. Dilutions from the pooled fractions were plated on CYE agar to determine bacterial CFUs. Data are the mean CFUs recovered from three independent wells±SD. Bacterial replication was calculated by determining the fold increase in CFUs.

### Single cell immunofluorescence assays to measure *L. pneumophila* uptake and formation of vacuoles containing replicating bacteria (RV)

*L. pneumophila* uptake and intracellular growth in both *Casp3^−/−^* and *Casp3^+/+^* DCs was performed as previously described [41]. Intracellular replication in B6, *Bak^−/−^*, and *Bax^−/−^Bak^−/−^* DCs was performed following the same protocol described previously with some modifications to the immunofluorescence staining [41]. Briefly, after permeabilization for 15 min at room temperature (R.T.) in RPMI containing 0.05% saponin, coverslips were incubated for 1 h at R.T. in permeabilization solution containing anti-MHC II I-A^b+d+q^, I-E^d+k^ antibody (TIB 120; American Type Culture Collection (ATCC), Rockville, MD). Coverslips were washed 3× in RPMI containing 0.05% saponin. Coverslips were incubated 45 min at R.T. with Alexa Fluor 568- conjugated goat anti-rat (Invitrogen-Molecular Probes) in permeabilization solution and then washed 3× with PBS. Coverslips were mounted on slides and examined by fluorescence microscopy. TIB 120 staining of MHC II was used to identify DCs. Assays to measure uptake and formation of vacuoles containing replicating *L. pneumophila* in BMMs were conducted similarly [14]. Data are represented by the mean number of cells observed in three independent coverslips.

### TUNEL staining

DCs previously selected by CD11c magnetic beads were infected with *L. pneumophila* at an MOI of 25 or treated for 5 h with staurosporine (1 µg/ml) and assayed for nuclear DNA fragmentation by TUNEL with the *in situ* cell death detection kit (Roche). Samples were then analyzed by fluorescence microscopy and all data points represent the average number of TUNEL positive cells±SD obtained from three independent coverslips.

### Immunoblotting

BMMs and DCs were directly lysed in SDS-PAGE sample buffer. Lysates were separated by SDS-PAGE, and proteins were transferred (Wet Transfer Cell; Bio-Rad) at 100 V for 1 h to Immobilon P membranes (Millipore) in transfer buffer (50 mM Tris, 40 mM glycine, and 10% methanol). Membranes were blocked for 1 h at 25°C in Tris-buffered saline (TBS), 5% nonfat dry milk, and 0.1% Tween-20. Membranes were incubated with primary antibody overnight at 5°C and incubated with horseradish peroxidase-conjugated secondary antibody 1 h at R.T. Rabbit anti-human Bcl-2, rabbit anti-Bax, and rabbit anti-Bak (Cell Signaling Technology) were used. Western Lightning Chemiluminescence Reagent Plus (Perkin Elmer) was used for antibody detection.

### Caspase-3/7 activity

Macrophages and DCs were plated in 96 well plates at a concentration of 5×10^4^ cells/well. Cells were infected with *L. pneumophila* at an MOI of 50, incubated at 37°C for 1, 2, 4, 6 and 11 hours and then frozen at −20°C to lyse the cells. Caspase-3/7 activity was measured using the Apo-One Homogeneous caspase-3/7 kit (Promega). Relative fluorescence units (RFU) measured at each time point is proportional to the amount of caspase-3/7 activity. All data points represent the average values±SD obtained from three wells assayed independently.

### Gene ID numbers

MyD88: 17874; Rip2: 192656; Caspase-1: 12362; Caspase-3: 12367; Bax: 12028; Bak: 12018; Human Bcl-2: 596; Naip5: 17951.

### Protein ID numbers

MyD88: P22366; Rip2: P58801; Caspase-1: P29452; Caspase-3: P70677; Bax: Q07813; Bak: O08734; human Bcl-2: P10415; Naip5: Q8CGT2.

## Supporting Information

Figure S1 Naip5-deficient DCs restrict *L. pneumophila* replication. Quantification of the percentage of *L. pneumophila* WT or Δ*dotA* infected B6 (black bars) and *Naip5^−/−^* DCs (white bars) with vacuoles containing replicating bacteria at 10 h post-infection. Data represent the mean±SD of 500 cells counted per coverslip in triplicate. R.V. = vacuoles containing replicating bacteria.
(0.46 MB EPS)

Figure S2 Nuclear fragmentation in DCs induced by *L. pneumophila* is caspase-1-independent. Quantification of the percentage of B6 (closed bars) and *Casp1^−/−^* DCs (open bars) infected with either *L. pneumophila* WT or Δ*dotA* that are TUNEL positive 6 h after infection. Data are represented by the mean±SD of 300 cells counted per each coverslip in triplicate.
(0.46 MB EPS)

Figure S3 *L. pneumophila*-induced activation of caspase-3/7 occurs faster in DCs compared to macropahges. (A) DCs and (B) BMMs were infected with either *L. pneumophila* WT or Δ*dotA* for 1 h, 2 h, 4 h, 6 h and 11 h as indicated. Caspase-3/7 activity is indicated as relative fluorescence units (RFU) measured at each time point. Data are expressed as mean±SD obtained from 3 independent wells. * p<0.05. **p<0.01.
(0.49 MB EPS)

Figure S4 *L. pneumophila* WT and Δ*flaA* replicate to similar levels in caspase-3-deficient DCs homozygous for the A/J Lgn1 allele. Intracellular replication of *L. pneumophila* WT, Δ*flaA* or Δ*dotA* was compared in *Casp3^−/−^* DCs at 36 h after infection. The fold increase in intracellular replication was determined by dividing *L. pneumophila* CFUs recovered at 36 h by the CFUs recovered at 1 h post infection. Data are the mean±SD from three independent wells. N.D. = not detectable.
(0.46 MB EPS)
